# Identification and Functional Analysis of miRNAs in the Cauda Epididymis of Yak and Cattle

**DOI:** 10.3390/ani16030492

**Published:** 2026-02-04

**Authors:** Dongju Liu, Linwen Ding, Xiaolong Yang, Xinyu Zhang, Xianrong Xiong, Yan Xiong, Jian Li, Duoji Gerong, Luobu Silang, Chengxu Li, Daoliang Lan, Shi Yin

**Affiliations:** 1 College of Animal & Veterinary Sciences, Southwest Minzu University, Chengdu 610041, China; qingjiu001020@163.com (D.L.); dlw19828447172@163.com (L.D.); yxlong0123@163.com (X.Y.); yu04end@163.com (X.Z.); xianrongxiong@163.com (X.X.); xiongyan0910@126.com (Y.X.); jianli_1967@163.com (J.L.); 2 Key Laboratory of Animal Science of National Ethnic Affairs Commission of China, Southwest Minzu University, Chengdu 610041, China; 3 Litang County Agriculture, Rural and Technology Bureau, Ganzi 627550, China; grdj1977@163.com (D.G.); ls19890122@126.com (L.S.); cllitang@163.com (C.L.)

**Keywords:** microRNA, cauda epididymis, yak, high-throughput sequencing

## Abstract

The yak represents a distinct domestic animal species that predominantly inhabits the Qinghai–Tibet Plateau and adjacent areas, possessing considerable value in both scientific and economic contexts. Compared to animals that mainly dwell on plains, such as cattle, the sperm maturation process in yak exhibits a certain degree of species specificity to adapt to their unique reproductive needs in high-altitude environments. Serving as the main storage site for functionally competent sperm, the cauda epididymis plays an integral role in mediating their post-testicular maturation. MiRNAs are vital regulatory molecules in the epididymis, influencing sperm maturation by modulating gene expression after transcription. To investigate the unique regulatory mechanisms of sperm maturation in yak, this study compared the miRNA expression profiles in the cauda epididymis of yak and cattle using high-throughput small RNA (sRNA) sequencing. The comparative analysis identified and characterized sRNA populations in the cauda epididymis of yak and cattle, revealing a similar length distribution that peaked at 22 nt and a predominance of known miRNAs. Notably, eight miRNAs were found to be highly expressed in both species. Furthermore, the first-nucleotide bias differed significantly between known and novel miRNAs within each species. A total of 31 differentially expressed (DE) miRNAs were identified, with 11 upregulated and 20 downregulated in yak compared to cattle. Among these, bta-miR-1298 exhibited the most significant upregulation, while bta-miR-2344 displayed the most pronounced downregulation. Bioinformatic analysis linked the predicted target genes of these miRNAs to numerous critical signaling pathways, including calcium signaling, the mitogen-activated protein kinase (MAPK) signaling pathway, the Ras-associated protein 1 (Rap1) signaling pathway, and the cyclic guanosine monophosphate-protein kinase G (cGMP-PKG) signaling pathway. Furthermore, eight significantly DE miRNAs, including bta-miR-2443, bta-miR-503-3p, bta-miR-6517, bta-miR-2440, bta-miR-2431-3p, bta-miR-2436-3p, bta-miR-6523a, and bta-miR-6775, were predicted to target genes involved in various aspects of sperm structural and functional maturation. These aspects include flagellum formation, sperm motility, chromatin remodeling, acrosome reaction, acrosome structure, sperm capacitation, chemotaxis, and nuclear chromatin condensation. Multiple miRNAs and their corresponding predicted target genes were analyzed by quantitative real-time PCR (qPCR), demonstrating an inverse correlation between miRNA expression and target gene levels. These findings reveal a distinct, species-specific miRNA signature in the yak cauda epididymis, which suggests a potential contribution to regulating the epididymal luminal environment and the process of sperm maturation. This study provides preliminary foundational data for elucidating the differences in sperm maturation mechanisms between yak and cattle, and offers potential novel targets for improving reproductive efficiency in plateau livestock.

## Simple Summary

Yak is an essential livestock species found on the Tibetan Plateau. Compared to animals that primarily inhabit plains, yak have a unique sperm maturation process tailored to their reproductive needs in high-altitude environments. The cauda epididymis is crucial for sperm development and is susceptible to post-transcriptional regulation by microRNAs (miRNAs). To investigate the regulatory dynamics of yak sperm maturation, miRNAs from this tissue in both yak and cattle were sequenced. A total of 497 miRNAs were identified across the two species. Comparative analysis revealed 31 miRNAs that were differentially expressed between them. Among these, 16 predicted target genes associated with eight miRNAs were found to be potentially linked to the structural or functional maturation of sperm. The expression of some miRNAs was validated, revealing an opposite trend compared to their corresponding predicted target genes. This study provides foundational data and new research perspectives for further understanding the gene regulatory network underlying yak sperm maturation in extreme environments.

## 1. Introduction

The epididymis serves as the crucial site where spermatozoa, after testicular production, acquire motility and fertilizing ability, thereby fulfilling its key functions in sperm storage, protection, and final maturation. Once sperm are produced in the testes, they move through the head and body of the epididymis before entering the cauda, where they attain full functional capacity [1,2,3]. The cauda epididymis acts as the primary reservoir for mature sperm, holding approximately 50–80% of the spermatozoa [1]. The epididymal fluid secreted by the epithelial cells in the cauda creates a unique microenvironment characterized by hypertonicity, low pH, and low oxygen tension. This specific physicochemical environment promotes sperm dehydration and tight compaction of nuclear chromatin. Additionally, it keeps sperm in a metabolically inactive state, reducing the generation of reactive oxygen species (ROS) and preserving sperm viability and energy reserves for their future journey [4,5,6]. The cauda epididymis is defined as the primary storage site for mature sperm, creating a unique physicochemical microenvironment that is integrally involved in the terminal maturation and extended maintenance of sperm motility and functional potential.

MicroRNAs function as important post-transcriptional regulators. These small (~22 nt) RNA molecules typically mediate gene silencing by base-pairing with target messenger RNAs, often within their 3′ untranslated regions. This connection either triggers the breakdown of the messenger RNA (mRNA) or blocks its translation into proteins, giving cells a sophisticated mechanism to keep gene expression in check [7,8]. Research has revealed that microRNAs serve as pivotal players in orchestrating a range of biological functions within the epididymis, encompassing cell division, ion channel regulation, and the enhancement of sperm movement [9,10,11]. For instance, through direct targeting and suppression of nuclear autoantigenic sperm protein (*NASP*), microRNA-29a curtails the proliferation of epididymal epithelial cells, fulfilling a key regulatory role in postnatal epididymal maturation [12]. The stable luminal environment of the epididymis is crucial for sperm maturation, with ions such as calcium (Ca^2+^) playing vital roles in critical events such as the acrosome reaction and capacitation [13]. The porcine epididymis has revealed that adrenoceptor beta 2 (*ADRB2*) and adenylate cyclase 3 (*ADCY3*) might be in the crosshairs of let-7a and miR-92a microRNAs, which both play pivotal roles in the intricate dance of Ca^2+^ and protein kinase A (*PKA*) signaling cascades [14]. Additionally, miR-26a regulates the glycolytic metabolic pathway by targeting pyruvate dehydrogenase complex component X (*PDHX*) in the porcine epididymis, ultimately affecting sperm motility in boars [15]. Thus, miRNAs present in the epididymis fulfill a pivotal and indispensable role in ensuring successful sperm maturation.

As a unique livestock species, the yak is predominantly native to the Qinghai–Tibetan Plateau and environs, where it holds economic importance. Compared to animals that primarily inhabit plains, such as cattle, the sperm maturation process in yak has adapted to high-altitude, cold, and hypoxic environments, allowing them to meet their specific reproductive needs under extreme conditions. These phenotypic differences may reflect distinct post-transcriptional regulation landscapes shaped by environmental adaptation. Significant interspecies differences in cauda epididymal miRNA expression profiles between yak and cattle may regulate divergent patterns of key gene expression during sperm maturation, which could contribute to observed variations in maturation efficiency and functional sperm competence. In this study, high-throughput sRNA sequencing was applied to comprehensively compare the miRNA expression profiles in the cauda epididymis between yak and cattle. The objective is to set up a basic miRNA database for future studies on reproduction and to elucidate the post-transcriptional control mechanisms affecting sperm development between yak and cattle. By elucidating the reproductive regulatory mechanisms underlying the yak’s adaptation to high altitude, this research aims to identify novel targets that could enhance reproductive efficiency in plateau livestock.

## 2. Materials and Methods

### 2.1. Animal and Tissue Samples

From three healthy adult male yaks and three cattle (aged 3–4 years) with comparable body weights, cauda epididymis samples were obtained at a slaughterhouse in Qingbaijiang District, Chengdu, Sichuan Province. Immediately after slaughter, the testis–epididymis complex was extracted, and the caudal segment of the epididymis was isolated. The tissues were washed with a phosphate-buffered saline (PBS) solution with 1% penicillin–streptomycin (Biosharp, Hefei, China, BL505A) before being submerged in RNA later (Yeasen, Shanghai, China, 10604ES60) for preservation. Following collection, all samples were immediately flash-frozen in liquid nitrogen and subsequently stored at −80 °C until analysis. The animal experiments were conducted in accordance with the guidelines approved by the Animal Ethics Committee of Southwest Minzu University (Approval No. SWU-202501231).

### 2.2. sRNA Library Construction

Following extraction with Trizol™ reagent (Invitrogen, Waltham, MA, USA, 15596026) from the cauda epididymis of yak and cattle, total RNA underwent integrity assessment by agarose gel electrophoresis to rule out DNA contamination. RNA purity was assessed by measuring the 260/280 nm absorbance ratio on a BioSpec-nano UV–Vis spectrophotometer (Shimadzu, Kyoto, Japan). Total RNA served as the starting material for library construction, leveraging the characteristic structural features of sRNAs—namely, a 5′-terminal phosphate group and a 3′-terminal hydroxyl group. Following this, adapters were ligated directly to both ends of the sRNAs, and the resulting molecules were subjected to reverse transcription to generate cDNA. The target DNA fragments were amplified via PCR. Subsequently, PCR products were separated using PAGE electrophoresis, and the target DNA fragments were excised and purified to construct the sRNA cDNA library. The AMPure XP Beads for DNA Cleanup (Beckman, Brea, CA, USA, A63881) was employed to purify the PCR products prior to sequencing. The base-calling error probability per base was calculated to assess the sequencing sample quality using FastQC (version 0.12.0) and the Phred quality score.

### 2.3. Clustering, Ranking, and Quality Control (QC)

Following library construction, index-coded samples were clustered using a cBot Cluster Generation System with the TruSeq PE Cluster Kit v4-cBot-HS (Illumina, San Diego, CA, USA), adhering to the manufacturer’s protocol. Subsequently, the prepared libraries were subjected to sequencing on an Illumina platform to generate single-end reads. Following initial generation, the raw sequence reads in FASTQ format underwent a rigorous quality-control pipeline. Through a series of meticulous processing steps, various problematic elements, including adapter sequences, Poly-N stretches exceeding 10%, and reads compromised by poor quality metrics (defined as bases with Q-scores of 5 or below constituting more than half of the sequence) were filtered out. This purification process, executed via custom Perl scripts in conjunction with the cutadapt tool, yielded high-quality clean reads ready for downstream analysis. Additionally, reads with 5′-adapter contamination, those lacking a 3′-adapter or insert fragment, and those consisting solely of poly-A/T/G/C were also discarded. For further analysis, a size selection was applied to retain sRNA sequences measuring 18–35 nt in length. The resulting clean datasets were first assessed for quality based on key metrics (Q20, Q30, error rate, and GC content) and then utilized as the basis for all subsequent bioinformatic analyses.

### 2.4. Annotation of sRNAs and Identification of miRNAs

To annotate sRNA sequences and identify miRNAs, the clean reads were aligned sequentially against multiple reference databases using Bowtie (version 1.0.1) [16]. First, the reads were compared to the Rfam database to identify and filter out non-coding RNAs. This filtration targeted known classes such as ribosomal RNA (rRNA), transfer RNA (tRNA), small nuclear RNA (snRNA), and small nucleolar RNA (snoRNA). Subsequent alignments against the Repbase database facilitated the identification of repetitive sequences. A hierarchical classification system was implemented to assign unique annotations, which prioritize known miRNA > rRNA > tRNA > snRNA > snoRNA > repeat > gene > novel miRNA. Unprocessed reads were aligned to the reference genome (*Bos taurus* ARS-UCD2.0, Ensembl 115) to generate mapped sequences. The aligned reads were then matched against established mature miRNA entries in the miRBase repository, allowing for sequence variations within a range of 2 nt upstream to 5 nt downstream, and permitting up to one mismatch. Reads that met these criteria were classified as known miRNAs. For the prediction of novel miRNAs, the unmapped reads were analyzed using the miRDeep2 (version 2.0.1.2) and miREvo (version 1.0) software, which evaluated characteristic precursor hairpin structures, Dicer enzyme cleavage sites, and minimum free energy parameters to identify putative novel miRNAs.

### 2.5. Identification of DE miRNAs

Transcripts per million (TPM) was employed to normalize and express the abundance of miRNAs. For datasets with biological replicates, differential expression analysis was conducted using DESeq2 (version 1.42.0), which applies a false discovery rate correction. Significantly DE miRNAs were identified using the following thresholds: |log_2_ fold change| ≥ 0 and adjusted *p*-value (*p*adj) < 0.05.

### 2.6. Functional Annotation of miRNA Target Genes

The miRNA targets were predicted using the miRanda (version 3.3a) and RNAhybrid (version 2.0) algorithms. On this basis, the identified miRNA-target relationships were utilized to perform Kyoto Encyclopedia of Genes and Genomes (KEGG) pathway enrichment analyses separately for the target gene sets of each DE miRNA group.

### 2.7. Quantitative Real-Time PCR for miRNA Quantification

Following RNA extraction with Trizol, cDNA was reverse-transcribed using a stem-loop miRNA-specific cDNA synthesis kit (Vazyme, Nanjing, China, MR101-01) as per the instructions. Primers were designed using NCBI resources and synthesized by Tsingke Biotechnology Co., Ltd. (Beijing, China). The primer sequences are detailed in Table S1. The qPCR assay was carried out in a 20 µL reaction volume comprising 10 µL of 2× miRNA Unimodal SYBR qPCR Master Mix (Vazyme, Nanjing, China, MQ102-01), 1 µL of cDNA template, 0.5 µL each of forward and reverse primers (10 µM concentration), and 8 µL of nuclease-free ddH_2_O to bring the total volume to completion. The thermal cycling was performed using a LightCycler 96 system (Roche, Basel, Switzerland, 05815916001). The amplification protocol kicked off with an initial denaturation phase at 95 °C for 30 s, followed by 40 cycles consisting of denaturation at 95 °C for 10 s and a combined annealing/extension step at 60 °C for 30 s. Relative expression levels were determined using the 2^−ΔΔCT^ method, with RNA, U6 Small Nuclear 1 (*U6*) serving as the internal control gene for data normalization.

### 2.8. Quantitative Real-Time PCR for mRNA Quantification

The HiScript III RT SuperMix for qPCR (+gDNA wiper) (Vazyme, Nanjing, China, R323-01) was employed to synthesize cDNA in accordance with the provided instructions. All primer sequences used were provided in Table S1. The qPCR was performed in a 20 µL reaction mixture comprising 10 µL of ChamQ Universal SYBR qPCR Master Mix (Vazyme, Nanjing, China, Q711-02), 1 µL of cDNA, 0.5 µL each of forward and reverse primers (10 μM), and 8 µL of nuclease-free ddH_2_O, then was performed on a LightCycler 96 instrument. The amplification protocol consisted of an initial denaturation at 95 °C for 1 min. This was followed by 40 cycles, each comprising a denaturation step at 95 °C for 10 s and annealing at 59 °C for 30 s, and extension at 72 °C for 30 s. Glyceraldehyde-3-phosphate dehydrogenase (*GAPDH*) was used as the reference gene, and the fold change in gene expression was quantified using the comparative CT (2^−ΔΔCT^) method.

### 2.9. Statistical Analysis

All experiments included a minimum of three independent replicates, and the results are expressed as the mean ± standard error of the mean (SEM). The statistical computations were performed using GraphPad Prism software (version 10.5). To assess the disparities between the two groups, a Student’s *t*-test was employed, and any difference deemed statistically meaningful was marked by a *p*-value < 0.05 (*p* < 0.05).

## 3. Results

### 3.1. Quality Evaluation and miRNA Identification of sRNA Sequences in the Cauda Epididymis of Yak and Cattle

Quality assessment of the sRNA sequencing data from the caudal epididymis of yak and cattle indicated good RNA integrity, with the RNA integrity number (RIN) values across all samples falling between 7.5 and 8.5. The number of raw reads varied from 11.36 to 13.34 million, with an average clean read ratio of 97.56% post-quality control, demonstrating high data quality. The GC content ranged from 49.61% to 51.64%, and the average Q30 base ratio was 97.91%, indicating high sequencing accuracy. The main contaminants filtered out included adapter contamination, low-quality reads, and sequences containing polyA/T/G/C, with the total proportion of filtered reads being less than 3.2% in all samples (Table 1). The findings validate the exceptional quality of the sequencing data, making them ideal for the subsequent analysis.

**Figure 1:**
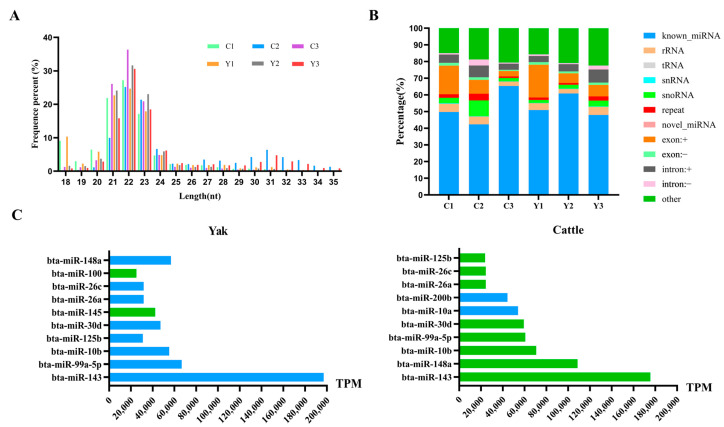
Identification of miRNAs in the cauda epididymis of yak and cattle. (**A**) sRNA length distribution. (**B**) Annotation and classification of sRNAs. (**C**) Top 10 most abundant miRNAs. Among these, the miRNA indicated by the green bar was highly expressed in both species, whereas those denoted by blue bars exhibited high expression exclusively in either yak or cattle.

In the cauda epididymis of cattle and yak, sRNA length distribution displayed a prominent enrichment within the 21–23 nt range, accounting for 68.90% and 69.47% of the total reads, respectively. The peak abundance occurred at 22 nt, representing 29.66% and 28.9% of the total reads in cattle and yak, respectively (Figure 1A). Annotation of the sRNAs revealed that known miRNAs accounted for the largest proportion, comprising 53.29% in yak and 52.48% in cattle, while the remaining fraction consisted of novel miRNAs and other RNA categories, including rRNA, tRNA, snRNA, snoRNA, repeats, and fragments derived from exons/introns (Figure 1B). Among the top 10 abundant miRNAs in each species, eight miRNAs, including bta-miR-143, bta-miR-99a-5p, bta-miR-148a, bta-miR-10b, bta-miR-30d, bta-miR-26a, bta-miR-26c, and bta-miR-125b, were highly expressed in yak and cattle. Additionally, bta-miR-100 and bta-miR-145 were highly expressed specifically in the caudal epididymis of yak, while bta-miR-200b and bta-miR-10a were highly expressed in the caudal epididymis of cattle (Figure 1C). These results provide a comparative overview of miRNA composition and abundance in the caudal epididymis of yak and cattle.

### 3.2. Base Performance Analysis

**Figure 2:**
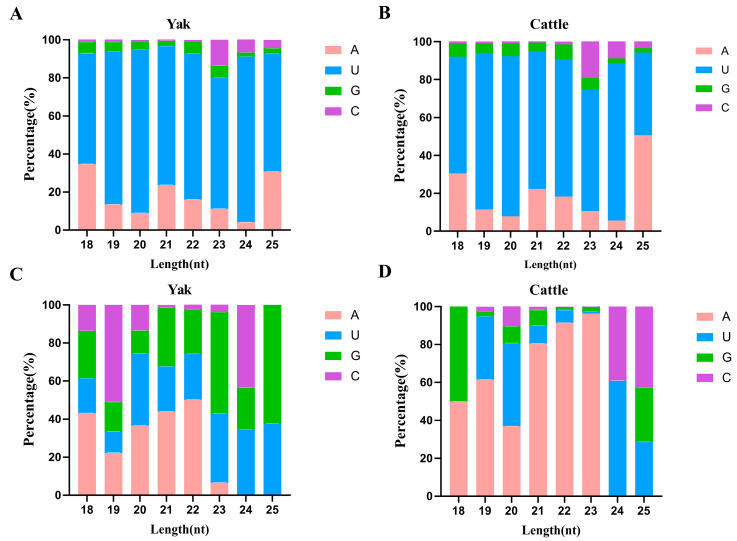
Nucleotide composition of 18–25 nt miRNAs in the cauda epididymis of yak and cattle. (**A**,**B**) First nucleotide bias performance of known miRNAs in yak and cattle. (**C**,**D**) First nucleotide bias performance of novel miRNAs in yak and cattle.

Analysis of nucleotide composition of miRNAs within the 18–25 nt range revealed distinct base preferences at the first position. Known miRNAs in yak and cattle exhibited a strong preference for uracil (U) at lengths 18–24 nt, at frequencies of 75.71% (yak) and 74.40% (cattle), respectively (Figure 2A,B). For miRNAs of 25 nt in length, 62.11% of known miRNAs in yak demonstrated a U preference, while 30.73% displayed adenine (A) at the first base position. In contrast, for cattle, the proportions were 50.59% for A and 43.58% for U. Analysis of nucleotide preferences in novel miRNAs revealed distinct patterns between yak and cattle (Figure 2C,D). In yak, A was the most frequent nucleotide at 18 nt (43.18%) and across the 20–22 nt lengths (43.56%), whereas cytosine (C) was predominant at 19 nt (51.11%). Guanine (G) was the preferred nucleotide at 23 nt (53.12%) and 25 nt (62.50%). Conversely, in novel miRNAs from cattle, A was strongly preferred at lengths 18–19 nt (51.00% and 61.54%) and overwhelmingly so across 21–23 nt (89.44%). U was the most frequent nucleotide at 20 nt (43.56%) and 24 nt (60.87%), while C was preferred at 25 nt (42.86%).

### 3.3. DE miRNAs in the Cauda Epididymis of Yak and Cattle

**Figure 3:**
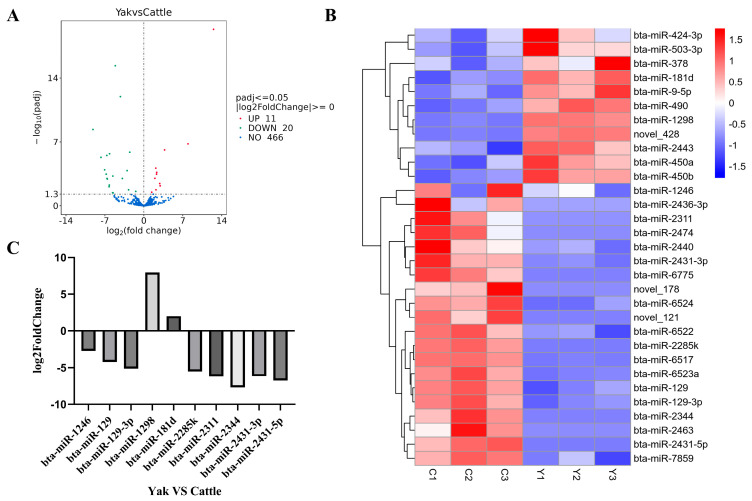
DE miRNAs in the cauda epididymis of yak and cattle. (**A**) Volcano plot illustrating the comparison of all miRNAs between yak and cattle. The Y-axis represents −log_10_(adjusted *p*-value) and the X-axis represents log_2_ fold change. Red dots depict upregulated miRNAs, green dots depict downregulated miRNAs, and blue dots represent non-significant miRNAs. (**B**) Heatmap displaying miRNA expression profiles across samples. Columns represent different samples, and rows represent different miRNAs. The clustering was based on the lg (TPM + 1 × 10^−6^) value. Color scale ranges from blue (low expression) to red (high expression), with white indicating intermediate levels. (**C**) Top 10 most significantly DE miRNAs. The Y-axis represents log_2_ fold change.

To investigate the potential functions of miRNAs in the cauda epididymis, a comparative analysis of miRNA expression profiles was conducted between yak and cattle, resulting in the identification of significantly DE miRNAs. A total of 31 DE miRNAs were identified between the two species, including 11 upregulated and 20 downregulated miRNAs in yak relative to cattle (Figure 3A, Table S2). A clustering heatmap of all DE miRNAs in the cauda epididymis of yak and cattle is also provided (Figure 3B). The Top 10 most significantly altered DE miRNAs were listed, with eight undergoing downregulation and two undergoing upregulation in the yak’s cauda epididymis. Notably, bta-miR-2344 exhibited the most drastic downregulation (log_2_ fold change = −7.71092) while bta-miR-1298 (log_2_ fold change = 7.941437) displayed the most significant upregulation (Figure 3C). To confirm the expression patterns of DE miRNAs, six DE miRNAs exhibiting distinct expression trends—including bta-miR-181d, bta-miR-490, bta-miR-6517, bta-miR-2433, bta-miR-503-3p, and bta-miR-2440—were meticulously chosen for expression profiling through qPCR. For all miRNAs analyzed, a high degree of correspondence was observed between the expression trends derived from qPCR and those from miRNA-seq, thereby validating the sequencing data (Figure S1).

### 3.4. Function Analyses of Predicted Target Genes Corresponding to DE miRNAs

**Figure 4:**
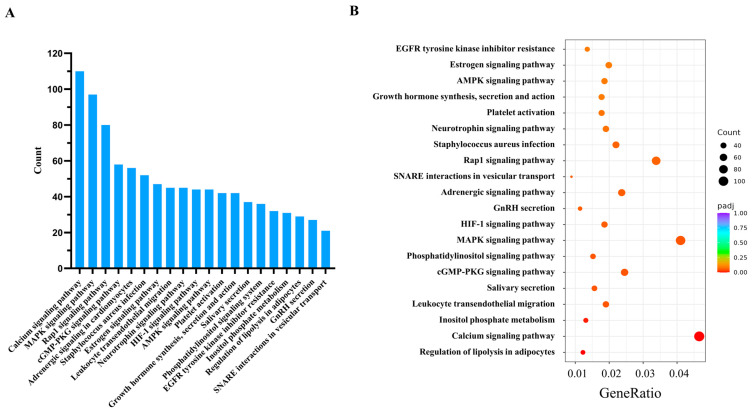
KEGG pathway enrichment analysis of target genes for DE miRNAs in the cauda epididymis of yak and cattle. (**A**) Bar plot displaying the Top 20 most significantly enriched pathways, with pathway names shown on the y-axis and the number of target genes annotated in each pathway indicated above the bars. (**B**) Bubble chart summarizing the enrichment results, where the y-axis lists pathway names and the x-axis represents the ratio of target genes mapped to a pathway relative to the total number of DE genes.

An in-depth functional enrichment analysis of the anticipated target genes linked to the DE miRNAs was performed via the KEGG database (Figure 4, Table S3). The Top 5 most significantly enriched KEGG pathways based on gene counts included the calcium signaling pathway (ID: bta04020), MAPK signaling pathway (ID: bta04010), Rap1 signaling pathway (ID: bta04015), cGMP-PKG signaling pathway (ID: bta04022), and adrenergic signaling in cardiomyocytes (ID: bta04261). Additionally, signaling pathways such as gonadotropin-releasing hormone (GnRH) secretion (ID: bta04929), growth hormone synthesis, secretion, and action (ID: bta04935), hypoxia inducible factor-1 (HIF-1) signaling pathway (ID: bta04066), AMPK signaling pathway (ID: bta04152), and soluble NSF attachment protein receptor (SNARE) interactions in vesicular transport (bta04130) were associated with hormonal regulation, metabolic modulation, and substance transport.

### 3.5. Identification of Target Genes of DE miRNAs Related to the Cauda Epididymis Between Yak and Cattle

**Figure 5:**
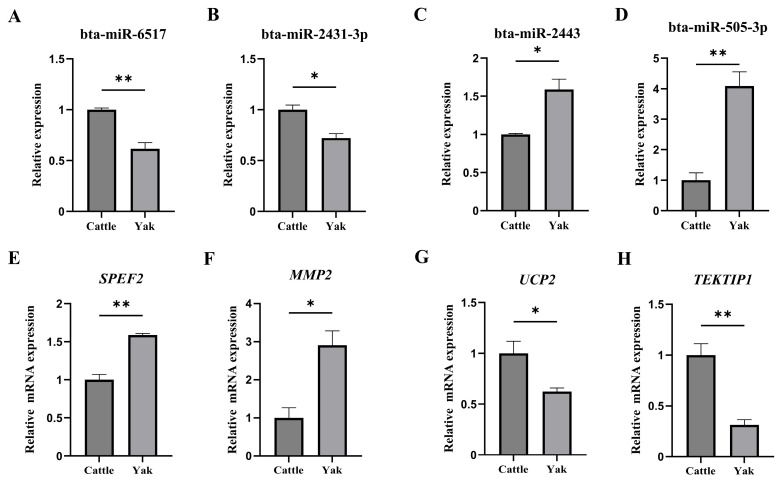
Expression detection of several DE miRNAs and their target genes related to spermatogenesis in the cauda epididymis of yak and cattle. (**A**–**D**) The levels of four distinct DE miRNAs—bta-miR-6517, bta-miR-2443, bta-miR-2431-3p, and bta-miR-505-3p—were measured and adjusted to the reference *U6*. (**E**–**H**) Expression detection of predicted target genes corresponding to the miRNAs in (**A**–**D**), including *SPEF2* (target of bta-miR-6517), *UCP2* (bta-miR-2443), *MMP2* (bta-miR-2431-3p), and *TEKTIP1* (bta-miR-503-3p), and adjusted to the reference *GAPDH*. Statistical significance at different thresholds was denoted as follows: * *p* < 0.05 and ** *p* < 0.01. Comparisons were analyzed using Student’s unpaired *t*-test.

For elucidating the functional roles of the DE miRNAs, their target genes were predicted and annotated (Table 2). Eight DE miRNAs corresponding to 18 predicted target genes were identified as being associated with sperm structural and functional maturation. These functions were classified into seven categories: Three genes (*MMP2*, *MFSD6L* and *NAPA*) involved in acrosome formation; seven genes (*IFT25*, *DNAH2*, *IFT20*, *TEKTIP1*, *DNAH1*, *TEKT1* and *SPEF2*) participated in flagellum formation; two genes (*WNT1* and *NEU1*) were associated with sperm capacitation; *NUP210L* and *ZCWPW1* were associated with chromatin remodeling; *NPPC* participated in chemotaxis; and *UCP2* was associated with sperm motility. Furthermore, four DE miRNAs, including bta-miR-6517, bta-miR-2443, bta-miR-2431-3p, and bta-miR-505-3p, together with some of their corresponding putative target messenger RNAs (mRNAs), were selected for expression validation using qPCR. The results revealed that bta-miR-6517 and bta-miR-2431-3p were significantly downregulated in yak compared with cattle, while bta-miR-2443 and bta-miR-505-3p were notably upregulated (Figure 5A–D). Correspondingly, the mRNA levels of their predicted target genes—*SPEF2* (target of bta-miR-6517), *MMP2* (target of bta-miR-2431-3p), *UCP2* (target of bta-miR-2443), and *TEKTIP1* (target of bta-miR-505-3p)—exhibited opposite trends (Figure 5E–H).

**Table 2:** DE miRNAs and their target genes involved in sperm maturation.

DE miRNAs	log_2_ Fold Change	*p*-Value	Predicated Target Genes and Their Functions
bta-miR-2431-3p	−6.167421736	1.83 × 10^−3^	*MMP2* (acrosome formation) [17]
bta-miR-2436-3p	−6.255443727	3.06 × 10^−4^	*IFT25* (flagellum formation) [18,19], *NPPC* (chemotaxis) [20], *WNT1* (sperm capacitation) [21]
bta-miR-2440	−3.011885148	1.94 × 10^−8^	*DNAH2* (flagellum formation) [22], *MFSD6L* (acrosome formation) [23]
bta-miR-2443	1.408315801	3.66 × 10^−6^	*IFT20* (flagellum formation) [24], *UCP2* (sperm motility) [25]
bta-miR-503-3p	2.929161932	3.28 × 10^−8^	*TEKTIP1* (flagellum formation) [26]
bta-miR-6517	−2.539893653	3.96 × 10^−4^	*ZCWPW1* (chromatin remodeling) [27], *DNAH1* (flagellum formation) [28], *TEKT1* (flagellum formation) [29], *SPEF2* (flagellum formation) [30], *NEU1* (sperm capacitation) [31], *NAPA* (acrosome formation) [32,33]
bta-miR-6523a	−6.658626156	5.67 × 10^−8^	*NUP210L* (chromatin remodeling) [34], *TEKT1* (flagellum formation) [29]
bta-miR-6775	−7.047914635	2.77 × 10^−6^	*DNAH2* (flagellum formation) [22]

## 4. Discussion

Spermatozoa maturity in the cauda epididymis is essential for acquiring fertilization capacity. This process involves complex gene regulation, with miRNAs acting as key factors in post-transcriptional regulation. This investigation examined miRNA expression patterns in the cauda epididymis of yak and cattle, offering valuable insights into the differences in sperm maturation mechanisms between these two species. The significantly differentially expressed miRNAs identified between two species may act as molecular markers for genetic breeding, facilitating the accelerated selection and breeding of plateau livestock. In addition, incorporating mimics of miRNAs that promote sperm motility or antagonists of miRNAs that inhibit sperm motility into semen extenders can help improve the quality of frozen semen and enhance the success rate of artificial insemination. Further research into the regulatory networks of key miRNAs may enable the development of targeted feed additives or pharmaceuticals, which could improve sperm maturation rates in breeding animals by modulating the levels of these miRNAs in vivo.

Analysis of miRNA nucleotide composition in the cauda epididymis of yak and cattle revealed a strong inclination toward U dominance at the initial nucleotide position (5′-end) of known miRNAs within the 18–24 nt length range in both species. This bias aligns with the sequence feature commonly observed in most animal miRNAs, which enhances the asymmetry of the miRNA/miRNA* duplex. This asymmetry promotes the preferential binding of the 5′-U-containing strand by Argonaut proteins, thereby ensuring accurate guide strand selection [35,36]. Notably, at 25 nt, the dominant 5′-terminal base shifted from U to A in cattle, while U remained predominant in yak. This divergence may reflect adaptive adjustments in post-transcriptional regulation networks along different evolutionary paths. The prevalent 5′-U preference is often associated with the functional conservation of miRNAs and the efficiency of target selection. However, under specific physiological conditions or in different tissue environments, nucleotide preferences can adapt, modulating the specificity and robustness of gene expression [37,38]. The sustained high U-bias in yak caudal epididymal miRNAs may help stabilize regulatory programs related to sperm maturation under hypoxic and cold-stress environments. The differing nucleotide preferences of novel miRNAs between the two species suggest species-specific origins or functions, indicating the need for further investigation into their biogenesis and physiological relevance.

Among all identified miRNAs, miR-143 was the most abundantly expressed in the cauda epididymis of both cattle and yak. Acting as a crucial form of post-transcriptional regulation, it influences fundamental cellular processes ranging from division and development to molecular signaling. According to prior studies, it influences dermal papilla cells in Hu sheep by suppressing proliferation, thereby extending the cell cycle duration [39]. Furthermore, it also promotes both angiogenesis and osteoblast differentiation, an effect mediated through its targeting of histone deacetylase 7 (*HDAC7*) [40]. Additionally, in ovarian granulosa cells, miR-143 mediates the follicle-stimulating hormone (FSH) signaling pathway, which in turn calls the shots on cell multiplication and estrogen production [41]. The conserved elevated expression of miR-143 points to a deep-rooted functional importance in the cauda epididymis. Investigating the balance between its conserved core functions and any species-specific regulatory targets will clarify how reproductive mechanisms are both maintained and adapted in yak and cattle.

The comparative profiling of miRNAs in the cauda epididymis of yak and cattle revealed 31 DE miRNAs. Among the Top 10 most significantly altered DE miRNAs, eight were notably downregulated in yak, while two were upregulated. MiR-1298 exhibited the most dramatic upregulation, while miR-2344 displayed the most pronounced downregulation. In the context of hypoxia/normoxia-induced myocardial injury in rats, miR-1298 was found to be abnormally expressed [42]. Its overexpression significantly elevated B-cell lymphoma-2 (*BCL2*), p-AMPK and phospho-glycogen Synthase Kinase-3β (p-GSK3β), reduced bcl-2-like protein 4 (*Bax*) levels, suppressed apoptosis, and preserved myocardial function [43]. Furthermore, in hypoxia-treated pulmonary arterial smooth muscle cells (PASMCs), miR-1298 was upregulated and mediated the bone morphogenetic protein/sma- and mad-related protein (BMP/Smad) signaling pathway, thereby alleviating the pulmonary hypertension phenotype [44]. Based on bioinformatic predictions, bta-miR-2344 potentially targets gap junction protein 3 (*GJB3*) and p21-activated kinase 4 (*PAK4*). Deficiency in *GJB3* expression impairs giant cell differentiation in mouse trophoblast stem cells, thereby abolishing their oxygen-sensing function [45]. In parallel, *PAK4* modulates the synthesis of hypoxia-inducible factor 1 alpha (*HIF-1α*) through the Akt-mTOR-4E-BP1 signaling cascade when oxygen levels are low [46]. Whether these DE miRNAs affect the structure or function of the cauda epididymis, thereby contributing to the observed differences in sperm maturation between the two species, requires further investigation.

KEGG pathway enrichment analysis of predicted target genes for DE miRNAs in the cauda epididymis revealed significant enrichment in several key pathways, such as calcium, MAPK, and Rap1 signaling. The calcium and MAPK signaling pathways are pivotal in the intricate regulation of sperm motility, capacitation, and the acrosome reaction, which are critical processes in the fertilization of the ovum. These pathways are not only essential for the viability and functionality of spermatozoa but also for the successful culmination of sexual reproduction in a vast array of organisms [47,48]. As a versatile secondary messenger, Ca^2+^ is central to sperm sensory signaling, transducing environmental cues into intracellular responses [49,50]. The enrichment of the calcium signaling pathway suggests that species-specific miRNA regulation may fine-tune intracellular Ca^2+^ homeostasis, potentially underlying the differences in sperm motility between yak and cattle. The MAPK/ERK signaling pathway plays a pivotal role in keeping the epididymal epithelium’s cells talking to each other and ensuring the tissue maintains its structural integrity [51]. Furthermore, this pathway is critically involved in modulating capacitation and post-testicular maturation through its downstream effectors [52]. The Rap1 pathway is reported to drive acrosomal calcium mobilization, which is essential for the acrosome reaction [53]. Moreover, the HIF-1 signaling pathway—the central regulatory pathway for cellular and systemic adaptation to hypoxia—was found to be enriched. This pathway helps maintain a hypoxic state in the cauda epididymis, ensuring low metabolic activity of sperm during storage [54]. It has also been reported that the HIF-1 pathway can enhance autophagy levels in sperm, thus facilitating the timely clearance of damaged or necrotic sperm in the cauda epididymis [55]. However, whether the predicted target genes corresponding to the differentially enriched miRNAs in this pathway directly contribute to the adaptive sperm maturation of yak in high-altitude environments, or whether only a correlation exists between them, requires further experimental validation in follow-up studies.

To elucidate the functional roles of DE miRNAs, their predicted target genes were characterized through bioinformatic annotation. Among the eight significantly DE miRNAs in the cauda epididymis between yak and cattle, six (bta-miR-2431-3p, bta-miR-2436-3p, bta-miR-2440, bta-miR-6517, bta-miR-6523a, and bta-miR-6775) were significantly downregulated in yak. The predicted target genes of these miRNAs were involved in flagellum formation (*IFT25*, *DNAH2*, *DNAH1*, *TEKT1*, and *SPEF2*), acrosome formation (*MMP2*, *MFSD6L*, and *NAPA*), chromatin remodeling (*NUP210L*), sperm capacitation (*WNT1* and *NEU1*), and chemotaxis (*NPPC*). Among them, two miRNAs, bta-miR-2431-3p and bta-miR-6517, and their corresponding predicted target genes, *SPEF2* and *MMP2*, were further validated to exhibit opposite expression trends. These results suggest that the cauda epididymis of yak may regulate sperm maturation by inhibiting the expression of these miRNAs. Compared with cattle, the cauda epididymis of yak must ensure the structural and functional maturation of sperm in extreme environments characterized by high altitude, hypoxia, and low temperatures. The differential miRNAs may finely regulate target genes to enable yak sperm to form a more stable chromatin structure, thus resisting oxidative damage caused by hypoxia while optimizing flagellum structure and motility patterns to enhance sperm chemotaxis and capacitation [17,18,19,20,21,22,23,24,25,26,27,28,29,30,31,32,33,34]. This allows for precise guidance through the female reproductive system and ensures sperm are ready to go at just the right moment, which significantly boosts the odds of successful fertilization when time is of the essence during mating. The functional tendencies of these differential miRNAs and their target genes likely imply a unique “regulatory program” in the epididymal microenvironment of yak. Through differential “fine-tuning” of key functional modules of sperm, this program endows sperm with physiological characteristics best adapted to the species-specific survival environment, mating behavior, and reproductive needs during maturation.

It should be noted that the selection of the well-annotated cattle genome was primarily intended to ensure consistency in cross-species alignment and to maximize the utility of existing miRNA databases, thereby facilitating direct comparisons with cattle and other reported Bos species studies. Due to genomic differences between yak and cattle, some miRNA precursors unique to yak or with substantial sequence variations may fail to align successfully, potentially leading to the omission of yak-specific miRNAs. Sequence polymorphisms could also reduce alignment efficiency, resulting in a systematic underestimation of the expression levels of certain miRNAs. Therefore, constructing or employing a high-quality yak genome for alignment is essential to obtain more comprehensive and accurate results in future research. In addition, although a few miRNA–target pairs were validated by qPCR, direct regulatory relationships and functional impacts on sperm maturation require further verification through functional assays in vitro or in vivo.

## 5. Conclusions

This research offers an initial comparative analysis of microRNA expression levels in the cauda epididymis of yaks and cattle, pinpointing a unique miRNA profile distinct to each species. These findings provide novel data and research perspectives for improving the breeds and enhancing the reproductive rates of livestock in plateau regions.

## Figures and Tables

**Table 1:** Evaluation of sequencing data.

Samples	RIN	Total Reads	Clean Reads	GC (%)	N% > 10%	5′ Adapter Contamine	3′ Adapter Null or Insert Null	With PloyA/T/G/C	Q30 (%)
C1	7.7	12,043,942	11,772,165	51.11	706	1733	257,224	12,114	98
C2	8.5	11,963,607	11,636,229	50.25	705	1610	306,656	18,407	97.9
C3	8.2	13,342,464	13,043,717	49.61	918	486	287,820	9523	98
Y1	7.5	11,362,627	11,093,687	51.64	771	1583	256,261	10,325	98.02
Y2	7.5	12,699,765	12,415,014	50.10	304	1019	275,430	7998	97.74
Y3	7.6	11,931,110	11,544,224	50.44	410	1169	373,463	11,844	97.78
Average	7.83	12,223,919	11,917,506	50.53	635.67	1266.67	2928.9	11,702	97.91

RIN: The RNA integrity number. Clean Reads: Number and percentage of reads retained after filtering. N% > 10%: Number and percentage of reads discarded due to ambiguous base (N) content exceeding 10%. 5′ Adapter Contamination: Number of reads filtered out due to containing 5′ adapter sequences and their proportion relative to total raw reads. 3′ Adapter or Insert Missing: Number of reads filtered out due to missing 3′ adapter or insert fragment, and their proportion relative to total raw reads. With Poly(A)/(T)/(G)/(C): Count and Proportion of Reads Filtered Due to Poly(A)/Poly(T)/Poly(G)/Poly(C) Sequences. Q30: Proportion of sequencing bases exceeding a Phred quality threshold of 30. Biological replicates for the cattle group were designated as C-1, C-2, and C-3, while those for the yak group were labeled Y-1, Y-2, and Y-3.

## Data Availability

The miRNA-seq raw data generated in this study are publicly accessible in the NCBI SRA database (Accession: SRR35803251-SRR35803256). All other supporting data are included in the published article and Supplementary Files. Requests for further details should be directed to the corresponding authors.

## Supplementary Materials

The following supporting information can be downloaded at: https://www.mdpi.com/article/10.3390/ani16030492/s1, Figure S1. The miRNA-seq data reliability verification. (A) Expression levels of six miRNAs randomly selected from the miRNA-seq dataset. (B) Validation of the miRNA expression levels shown in (A) by qPCR. * *p* < 0.05, ** *p* < 0.01, *** *p* < 0.001 indicated statistical significance at different levels; Student’s unpaired *t*-test. Table S1. Primer sequences. Table S2. Details of DE miRNAs in the cauda epididymis of yak and cattle. Table S3. The KEGG enrichment of DE miRNAs in the cauda epididymis of yak and cattle.

## Abbreviations

The following abbreviations are used in this manuscript:

miRNAs	microRNAs
sRNA	small RNA
DE	differentially expressed
MAPK	mitogen-activated protein kinase
Rap1	ras-associated protein 1
cGMP-PKG	cyclic guanosine monophosphate-protein kinase G
qPCR	quantitative real-time PCR
ROS	reactive oxygen species
mRNA	messenger RNA
NASP	nuclear autoantigenic sperm protein
Ca^2+^	calcium ions
ADRB2	adrenoceptor beta 2
ADCY3	adenylate cyclase 3
PKA	protein kinase A system
PDHX	pyruvate dehydrogenase complex component X
PBS	phosphate-buffered saline
QC	quality control
FASTQ	fast alignment sequence quality
rRNA	ribosomal RNA
tRNA	transfer RNA
snRNA	small nuclear RNA
snoRNA	small nucleolar RNA
TPM	transcripts per million
KEGG	Kyoto encyclopedia of genes and genomes
U6	RNA, U6 Small Nuclear 1
GAPDH	glyceraldehyde-3-phosphate dehydrogenase
SEM	standard error of the mean
RIN	RNA integrity number
U	Uracil
A	Adenine
C	Cytosine
G	Guanine
GnRH	gonadotropin-releasing hormone
HIF-1	hypoxia inducible factor-1
SNARE	soluble NSF attachment protein receptor
HDAC7	histone deacetylase 7
FSH	follicle-stimulating hormone
BCL2	B-cell lymphoma-2
p-GSK3β	phospho-glycogen Synthase Kinase-3β
BAX	bcl-2-like protein 4
PASMCs	pulmonary arterial smooth muscle cells
BMP/Smad	bone morphogenetic protein/sma- and mad-related protein
GJB3	gap junction proteins 3
PAK4	p21-activated kinase 4
HIF-1a	hypoxia inducible factor 1 subunit alpha
MAPK/ERK	mitogen-activated protein kinase/extracellular signal-regulated kinase
