# Supplementary figures and images for: Identification and Functional Analysis of miRNAs in the Cauda Epididymis of Yak and Cattle
Source: Animals (Basel). 2026 Feb 4;16(3):492. doi: 10.3390/ani16030492 (PMC12897380; doi:10.3390/ani16030492)

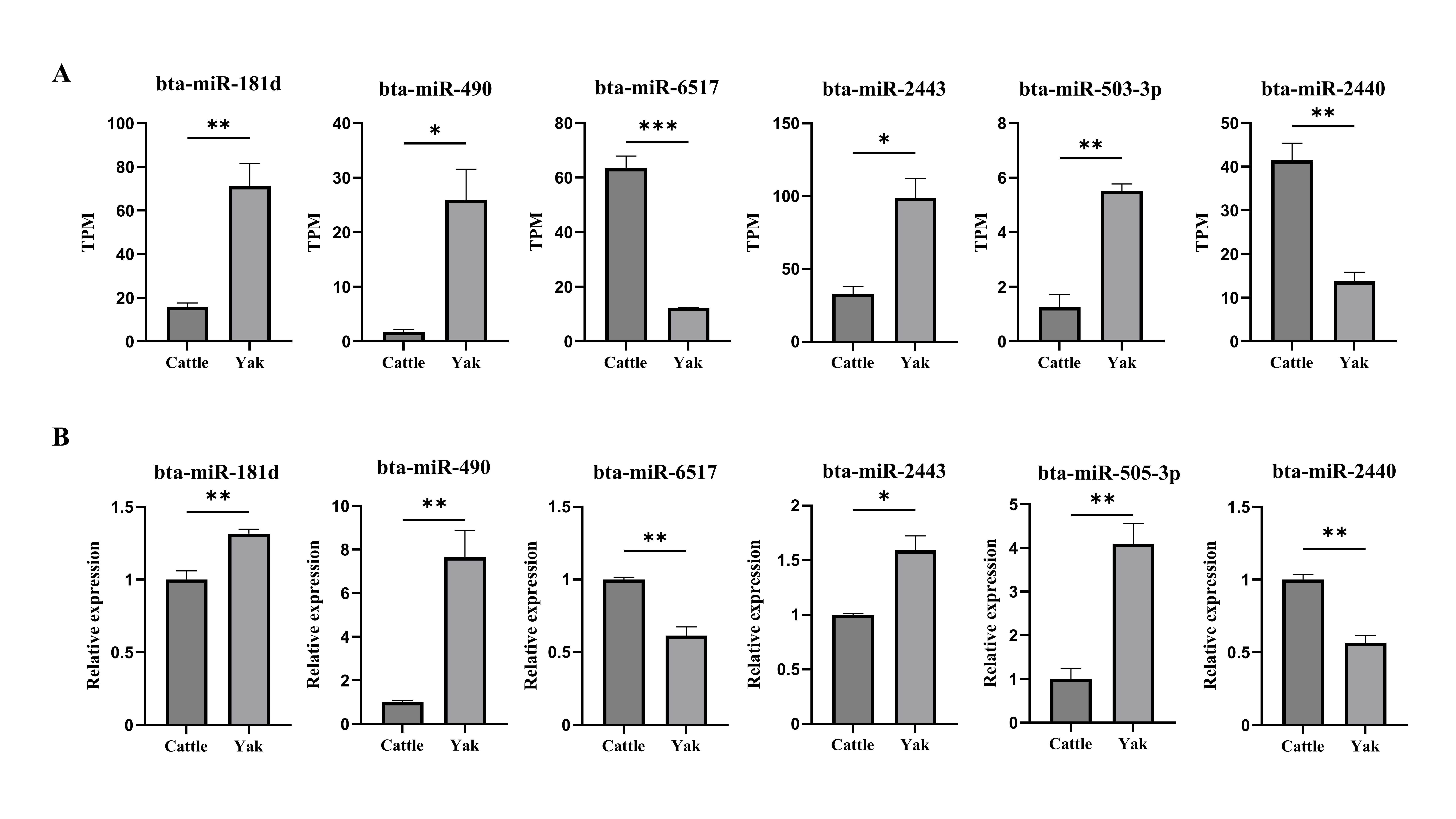

Supplement: Supplementary file 1 [file animals-16-00492-s001.zip › Fig S1.tif]
